# Novel Physique Index for the Screening of Skeletal Dysplasia at Birth

**DOI:** 10.3390/children8050331

**Published:** 2021-04-25

**Authors:** Ryoji Aoki, Nobuhiko Nagano, Aya Okahashi, Shoko Ohashi, Yoshinori Fujinaka, Itsuro Takigawa, Ken Masunaga, Ichiro Morioka

**Affiliations:** 1 Department of Pediatrics and Child Health, Nihon University School of Medicine, Oyaguchi-kamimachi, Itabashi-ku, Tokyo 173-8610, Japan; aoki.ryoji@nihon-u.ac.jp (R.A.); nagano.nobuhiko@nihon-u.ac.jp (N.N.); okahashi.aya@nihon-u.ac.jp (A.O.); 2 Department of Neonatology, Tokyo Metropolitan Ohtsuka Hospital, Tokyo 170-8476, Japan; shoko_ohashi@tmhp.jp (S.O.); yoshinori_fujinaka@tmhp.jp (Y.F.); simanerima@gmail.com (I.T.); ken_masunaga@tmhp.jp (K.M.)

**Keywords:** birth weight, head circumference to height ratio, femur length, newborn, small for gestational age

## Abstract

This study aimed to devise a novel physique index and investigate its accuracy in identifying newborns with skeletal dysplasia in comparison with head circumference (HC)/height (HT) ratio. The birth weight (W), HT, and HC at birth of 1500 newborns were retrospectively collected. The linear regression equations and coefficients of determination (R^2^) were determined. The formulated equation was corrected by the mean weight for gestational age at birth (Wcorr) as a novel physique index for screening skeletal dysplasia. The index accuracy was assessed using receiver operating characteristic (ROC) curves in 11 newborns by fetal ultrasound and compared with that of the HC/HT ratio. The R^2^ values between W and HT, (HT)^2^, and (HT) ^3^ were 0.978, 0.990, and 0.993, respectively. Those between W and HC, (HC)^2^, and (HC)^3^ were 0.974, 0.984, and 0.988, respectively. W/Wcorr × (HC/HT)^3^ was used as a novel physique index. Seven newborns had skeletal dysplasia. Our novel physique index had a higher area under the curve (AUC), sensitivity, and specificity than the HC/HT ratio (AUC: 1.00 vs. 0.86, sensitivity: 1.00 vs. 0.86, and specificity: 1.00 vs. 0.75, respectively). Our novel physique index was more accurate than HC/HT ratio and has the potential to accurately identify newborns with skeletal dysplasia.

## 1. Introduction

Skeletal dysplasia is a disorder of the development and growth of bones or cartilages [1]. The most recent classification of skeletal dysplasia includes 42 groups and 436 diseases [1]. Skeletal dysplasia may lead to several complications during infancy. For example, patients with achondroplasia often develop otitis media, obstructive apneic attack, hydrocephalus, or stenosis of the craniocervical spine junction, necessitating surgical interventions [2]. In particular, stenosis of the craniocervical spine junction may cause sudden death in infancy. Therefore, early diagnosis of skeletal dysplasia is important to prevent or treat these complications [3,4].

Skeletal dysplasia is accurately diagnosed by radiographic and genetic findings [1,5]. Before radiographic and genetic examinations, the newborn has to be suspected of having skeletal dysplasia by the attending clinicians. Some cases can be suspected by the disproportionate short limbs at birth. However, approximately 20% of achondroplasia cases are not diagnosed by visual inspection during infancy but diagnosed during early childhood [3]; because hypochondroplasia has fewer clinical characteristics in infancy than achondroplasia, it is often diagnosed by the presence of a short stature at the age of 2−3 years [6].

Objective and easy-to-use screening methods are needed for pediatricians or neonatologists. For screening of fetal skeletal dysplasia, a section of the femoral length (FL) is generally used during fetal ultrasound examination [7,8]. However, the accuracy of this method is limited. Todros et al. had reported that only 11 (13%) out of 86 cases could be diagnosed with skeletal dysplasia using an FL of <−2.0 standard deviation scores (SDSs) in the second trimester [7]. Likewise, Kruts et al. have reported that only 11 (46%) out of 28 fetuses with skeletal dysplasia had an FL of <−2.0 SDS [8]. Even when the FL cut-off value was set to <−4.0 SDS, the sensitivity was 83% (10 out of 12 fetuses) [8]. 

Because many patients with skeletal dysplasia have short stature and head circumference (HC) expansion, the HC/height (HT) ratio is used to screen for skeletal dysplasia only in children, but not in newborns [9,10]. In pediatric achondroplasia and hypochondroplasia, the HC/HT ratio is more prominent compared to HT or HC alone [10]. However, the HC/HT ratio is used for screening for macrocephaly or abnormal body proportions, and not skeletal dysplasia [9,10]. To date, no studies have evaluated the HC/HT ratio at birth as a screening tool for skeletal dysplasia in newborns. Furthermore, because there is no accurate screening method for skeletal dysplasia at birth, we aimed to devise a new physique index as an objective and easy-to-use screening for skeletal dysplasia at birth.

## 2. Materials and Methods

### 2.1. Study Design

Two studies were performed. In Study 1, a novel physique index for screening skeletal dysplasia was developed, and in Study 2, the index was validated. In Study 1, a cohort of 1500 newborns born in 2016 at Nihon University Itabashi Hospital and Tokyo Metropolitan Ohtsuka Hospital, Tokyo, Japan, was included. In Study 2, a single hospital-based retrospective study using the data of 11,146 newborns born between 2006 and 2016 at the Tokyo Metropolitan Ohtsuka Hospital, Tokyo, Japan was performed. 

### 2.2. Definitions

Shortening of limbs was defined as a fetal FL of <−3.0 SDS for gestational age just prior to birth using fetal ultrasound by registered and practicing obstetricians [7,8,11].

Skeletal dysplasia was diagnosed using bone X-rays by the consensus of two registered and practicing radiologists specialized in skeletal dysplasia. In cases 1 to 6, the separate bone X-rays for upper limbs, lower limbs, head, chest, vertebral body, and pelvis were taken. In case 7, the bone X-ray of the whole body was taken on a single view.

Genetic tests for skeletal dysplasia were performed only for patients whose parents/guardians consented to perform such analyses. The genetic analyses were kindly performed using polymerase chain reaction, direct sequence, and multiplex ligation-dependent probe amplification by the genetic specialists in their laboratories as previously described (see Acknowledgments) [12,13,14], and the mutations were confirmed.

Small for gestational age (SGA) was defined using the following criteria based on sex-specific Japanese standards [15]: (i) birth weight (W) and HT of <10th percentile and (ii) W and/or HT of <−2.0 SDS for gestational age at birth [16]. The SDSs for W, HT, and HC at birth were calculated using nordiFIT (Novo Nordisk Pharma, Tokyo, Japan).

### 2.3. Study Methods and Statistical Analyses

Study 1: The following data were retrospectively collected: W, HT, HC, sex, and gestational age at birth. The W was measured using an electric scale, and HT was measured by the crown-to-heel length using a measuring tape or ruler by midwives or trained nurses. The coefficients of determination (R^2^) were determined between W and HT, (HT)^2^, and (HT)^3^ and between W and HC, (HC)^2^, and (HC)^3^ using linear regression analyses. Using the parameters for HT and HC with the highest R^2^, the equation was formulated and then corrected by the mean weight for gestational age at birth (Wcorr). 

Study 2: Our physique index was validated using receiver operating characteristic (ROC) curves. The area under the curve (AUC), sensitivity, and specificity were compared with those of the HC/HT ratio in newborns who were suspected of having shortened limbs by fetal ultrasound examination. The cut-off value was determined using the maximum Youden index of the ROC curve. The Youden index is the point farthest from the boundary delineating the area under the curve (0.500 on the ROC curve) and represents the value of sensitivity + specificity – 1 [17]. All statistical analyses were performed using JMP version 14 (SAS Institute Inc., Tokyo, Japan) and Bellcurve for Excel version 3.20.

## 3. Results

### 3.1. Study 1

Of the 1500 newborns (744 (50%) boys and 756 (50%) girls), 832 (55%) and 668 (45%) were born to primiparous and multiparous mothers. In the modes of delivery, vaginal was 1097 (73%) and cesarean section was 403 (27%). The median gestational age at birth was 39 weeks (range, 22–41 weeks), the median W was 2935 g (range, 447–4935 g), the median HT was 47.8 cm (range, 27.4–57.2 cm), the median HC was 33.1 cm (range, 19.5–37.5 cm), and the median HC/HT ratio was 0.69 (range, 0.60–0.82).

**Figure 1:**
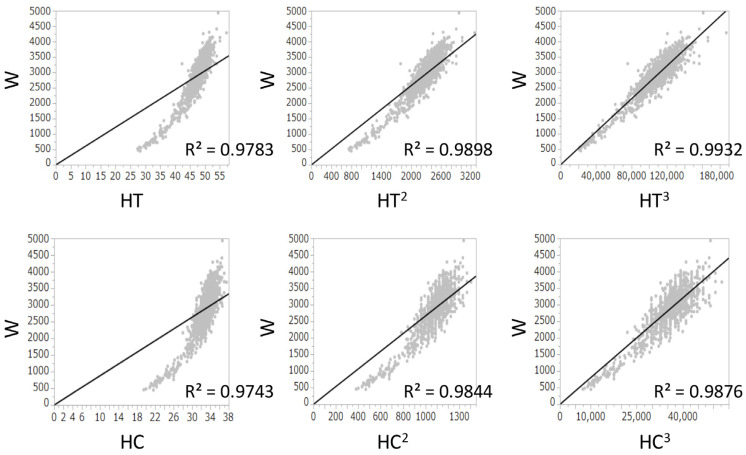
Linear regression analyses between birth weight and height, (height)^2^, or (height)^3^ and between birth weight and head circumference, (head circumference)^2^, or (head circumference)^3^ at birth. R^2^, coefficients of determination; HC, head circumference; HT, height; W, birth weight.

**Figure 2:**
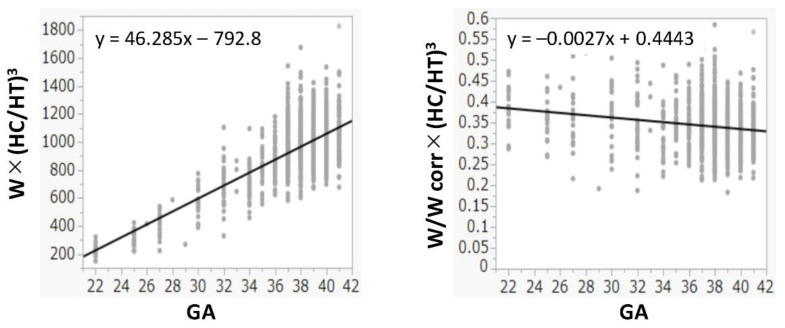
Physique index corrected by birth weight or birth weight corrected by mean weight for gestational age at birth. GA: gestational age at birth; HC: head circumference; HT: height; W: birth weight; Wcorr: mean weight for gestational age at birth.

The R^2^ values between W and HT, (HT)^2^, and (HT)^3^ were 0.978, 0.990, and 0.993, respectively. The R^2^ values between W and HC, (HC)^2^, and (HC)^3^ were 0.974, 0.984, and 0.988, respectively. Because both (HT)^3^ and (HC)^3^ had the highest R^2^ values and had a positive correlation with W (Figure 1), (HC/HT)^3^ was determined as the best formula, and W was multiplied by (HC/HT)^3^. Because the value of W × (HC/HT)^3^ was dependent on the gestational age, it was divided by Wcorr. The value of W/Wcorr × (HC/HT)^3^ was almost the same for all gestational ages (Figure 2). Finally, W/Wcorr × (HC/HT)^3^ was selected as the novel physique index.

### 3.2. Study 2

The number of newborns born during the study period was 11,146, of whom 11 (0.11%) had a fetal FL of <−3.0 SDS for gestational age. The detailed data of 11 newborns with a fetal FL of <−3.0 SDS for gestational age are shown in Table 1. Of the 11 newborns with a shortened FL, 7 (64%) had skeletal dysplasia. The final diagnosis of skeletal dysplasia was hypochondroplasia (*n* = 2), spondyloepiphyseal dysplasia congenita (*n* = 2), achondroplasia (*n* = 1), atelosteogenesis type III (*n* = 1), and thanatophoric dysplasia (*n* = 1). All infants with nonskeletal dysplasia were SGA (Table 1).

**Figure 3:**
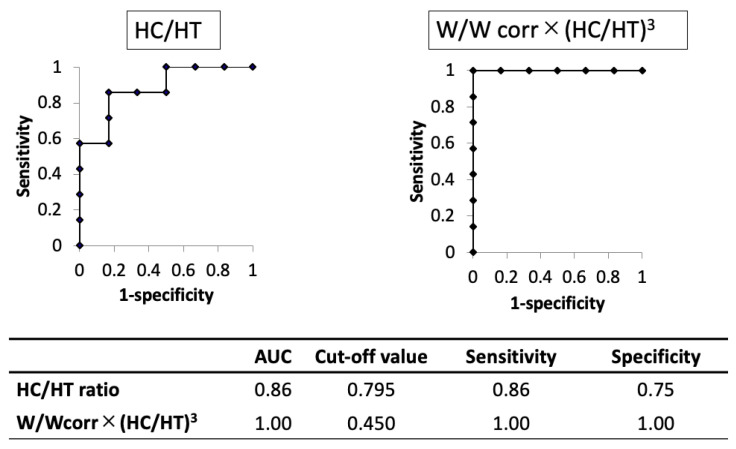
Comparison of the results of the receiver operating characteristic curve analysis between head circumference/height ratio and the novel physique index in cases with shortening of limbs in utero. AUC, area under the curve; HC, head circumference; HT, height; W, birth weight; Wcorr, mean weight for gestational age at birth.

The AUC of the novel physique index was higher than that of the HC/HT ratio (1.00 and 0.86, respectively). When using the cut-off value of 0.795 for the HC/HT ratio or 0.450 for the novel physique index, the sensitivity and specificity were 0.86 and 0.75 in the HC/HT ratio or 1.00 and 1.00 in the novel physique index, respectively (Figure 3).

## 4. Discussion

This study demonstrated two novel findings. The HC/HT ratio at birth was limited in screening for skeletal dysplasia. The novel physique index (W/Wcorr × (HC/HT)^3^), having a higher accuracy as compared to the HC/HT ratio, was developed for screening of skeletal dysplasia at birth.

To date, the HC/HT ratio is the only available index for the screening of skeletal dysplasia in children aged 0−5 years [10]. For the first time, the present study showed a median HC/HT ratio of 0.69 in 1500 Japanese newborn infants, which was similar to a median HC/HT ratio of 0.70 in 3571 Argentinian children aged 0−5 years [10]. Thus, the same HC/HT ratio may be used regardless of age (≤5 years) and ethnicity. The present study also clarified the accuracy of the HC/HT ratio for the screening of skeletal dysplasia in newborns with a fetal FL of <−3.0 SDS. This ratio was found to produce false-positive and false-negative results, even though the number of patients was limited. Therefore, a more accurate index is required.

Flechtner et al. reported that some cases of skeletal dysplasia were included in SGA children [18]. Of the 93 SGA children, 16 (17%) were diagnosed with skeletal dysplasia and 17 (18%) had minor skeletal abnormalities. They also reported that 6 of 17 children with achondroplasia were SGA [18], indicating that the differentiation between SGA and skeletal dysplasia is very important. Further, it is difficult to screen for skeletal dysplasia based only on body weight and HT; therefore, we devised a novel physique index using the W and HC/HT ratios.

For the assessment of physique in children depending on the age, the body mass index and the Kaup index use body weight divided by height squared, while the Laurel index uses body weight divided by height cubed [19]. In newborns, the body weight is generally proportional to the height cubed [20], which is consistent with our present results (Figure 1). In addition, we also found that the HC cubed and W had the best correlations (Figure 1). In adults, the actual body weight divided by the ideal weight is used to assess obesity and leanness [21]. Similarly, W should be corrected by the mean (ideal) W based on the gestational age at birth, as used in the definition of SGA [15,22]. Hence, correction by the Wcorr produced the physique index. Its value was almost constant in all newborns, regardless of the gestational age at birth (Figure 2).

In an observational study using an FL cut-off of <−2.0 SDS for gestational age, the false-positive rate for detecting skeletal dysplasia was 87% [7]. In a study comparing 16 fetuses with an FL between −2.0 SDS and −4.0 SDS and 12 fetuses with an FL of <−4.0 SDS, the false-positive rates for skeletal dysplasia were 100% and 17%, respectively [8]. Because false-positive results are more frequent when using an FL of <−2.0 SDS, a Japanese obstetric guideline mentioned that an FL of −3.0 to −4.0 SDS should raise the suspicion of skeletal dysplasia [23]. Hence, in this study, limb shortening was defined as a fetal FL of <−3.0 SDS for gestational age. Of the 11 newborns with an FL of <−3.0 SDS, 7 had skeletal dysplasia as we expected. Our novel physique index detected all newborns with skeletal dysplasia (false-positive rate: 0%). Two cases of hypochondroplasia were included, suggesting that it can detect skeletal dysplasia in newborns without clinical characteristics in the neonatal period [6].

There were some limitations to our study. The sample size of the validation study was smaller because of the rarity of cases with skeletal dysplasia and severe SGA with an FL of <−3.0 SDS for gestational age. Moreover, this was an observational study performed in a single Japanese hospital. The utility of the index could not be examined in all diseases under skeletal dysplasia. Further multicenter studies including a large cohort of skeletal dysplasia should be conducted.

## 5. Conclusions

In conclusion, the novel physique index developed in this study was found to be more accurate compared to the HC/HT ratio and has the potential to accurately identify newborns with skeletal dysplasia. Further validation studies using a large cohort of newborns with skeletal dysplasia are required to evaluate the utility of this index.

## Figures and Tables

**Table 1:** Detailed data of 11 newborns with a fetal femoral length of <−3.0 standard deviation score for the gestational age.

A. Physique							
Case	SDS of Fetal FL (GA at Measurement)	GA at Birth	W (g)	HT (cm)	HC (cm)	HC/HT	W/Wcorr × (HC/HT)^3^
1	−4.3 (36 weeks)	39 weeks 0 days	3088	46.0	35.0	0.76	0.45
2	−3.9 (30 weeks)	33 weeks 1 day	2000	41.3	33.2	0.80	0.53
3	−4.4 (36 weeks)	37 weeks 6 days	3068	45.5	36.2	0.80	0.57
4	−6.0 (38 weeks)	39 weeks 2 days	2988	41.3	35.5	0.86	0.63
5	−6.8 (36 weeks)	37 weeks 0 days	2570	40.5	37.2	0.92	0.75
6	−7.8 (37 weeks)	38 weeks 2 days	2612	37.0	34.2	0.92	0.72
7	−11.4 (36 weeks)	36 weeks 4 days	1762	31.0	32.7	1.10	0.80
8	−3.2 (27 weeks)	27 weeks 2 days	680	32.1	25.4	0.79	0.32
9	−5.1 (30 weeks)	30 weeks 6 days	684	32.2	26	0.81	0.23
10	−3.4 (39 weeks)	39 weeks 5 days	2010	44.2	30.5	0.69	0.21
11	−3.4 (35 weeks)	36 weeks 2 days	2014	43.1	31.5	0.73	0.31
**B. Radiological and Genetic Findings**							
**Case**	**Radiological Findings**				**Genetic Findings**		**Final Diagnosis**
1	Large calvarium, dorsal scalloping of vertebral bodies, horizontal acetabular margins, narrow sciatic notch, short tubular bones, oval radiolucent area in the proximal femur, cone-shaped middle phalanges, and trident hand				*FGFR3* c.1138 G>A p.Gly380Arg		Achondroplasia
2	Protuberance of frontal bones, small skull base, horizontal acetabular margins, narrow sciatic notch, mild short tubular bones, cone-shaped middle phalanges, and trident hand. No evidence of dorsal scalloping of vertebral bodies and metaphyseal flaring of the distal femur				NE		Hypochondroplasia
3	Protuberance of frontal bones, horizontal acetabular margins, narrow sciatic notch, mild short tubular bones, cone-shaped middle phalanges, and trident hand. No evidence of metaphyseal flaring of the distal femur				*FGFR3* c.1620C>A p.Asn540Lys		Hypochondroplasia
4	Lack of ossification of the os pubis, distal femur, and proximal tibial epiphyses. Pear-shaped vertebra, short iliac bones in craniocaudal dimension, and horizontal acetabular margins				*COL2A1* c.5404del		Spondyloepiphyseal dysplasia congenita
5	Lack of ossification of the os pubis, distal femur, and proximal tibial epiphyses. Pear-shaped vertebra and horizontal acetabular margins				*COL2A1* c.2094G>A p.Gly699Asp		Spondyloepiphyseal dysplasia congenita
6	Small vertebral bodies and coronal clefts throughout the thoracic and lumbar spine, flared iliac wings, steep acetabula, and disproportionately short and distally tapered humerus and femur				*FLNB* c.650C>T p.Pro217Leu		Atelosteogenesis type III
7	Narrow thorax due to a short rim; distinct flatness of the ossification centers of the vertebral bodies; horizontal inferior margins of the iliac bones; and very short, broad, and bowed long tubular bones				NE		Thanatophoric dysplasia
8	Normal				NE		SGA
9	Normal				NE		SGA
10	Normal				NE		SGA
11	Normal				NE		SGA

FL, femoral length; GA, gestational age; HC, head circumference; HT, height; NE, not examined; SDS, standard deviation score; W, weight; Wcorr; weight corrected by the mean weight for the gestational age.

## Data Availability

The data presented in this study are available on request from the corresponding author.
